# Identifying Suitable Regions for *Fritillaria unibracteata* Cultivation Without Damage from the Pest *Eospalax baileyi*

**DOI:** 10.3390/plants14050674

**Published:** 2025-02-22

**Authors:** Changrong Deng, Jianling Li, Shan Tao, Yuan Jin, Fang Peng

**Affiliations:** 1 State Key Laboratory of Plateau Ecology and Agriculture, Qinghai Key Laboratory of Vegetable Genetics and Physiology, Qinghai Plateau Key Laboratory of Tree Genetics and Breeding, Laboratory for Research and Utilization of Qinghai Tibet Plateau Germplasm Resources, Academy of Agriculture and Forestry Sciences, Qinghai University, Xining 810016, China; dengchang_rong@126.com (C.D.); lijianling@qhu.edu.cn (J.L.); 2 Industrial Crop Research Institute, Sichuan Academy of Agricultural Sciences, Chengdu 610300, China; jzstaoshan@163.com; 3 Ecological Protection and Development Research Institute of Aba Tibetan and Qiang Autonomous Prefecture, Wenchuan 624099, China; jy8883292@163.com

**Keywords:** *Fritillaria unibracteata*, *Eospalax baileyi*, MaxEnt, geographical distribution, pest management

## Abstract

The plateau zokor, *Eospalax baileyi* Thomas, is a destructive mammal pest affecting the cultivation of the medicinal plant *Fritillaria unibracteata* Hsiao et K.C. Hsia. Identifying regions exclusively suitable for the plant is an effective way to mitigate zokor-induced damage. In this study, the optimal MaxEnt model and ArcGIS were employed to predict suitable habitats for both species and identify pest-free regions for plant cultivation. Our results indicate that elevation and annual mean temperature are the critical factors influencing the plant distribution, while the pest distribution is determined by the elevation and precipitation of t warmest quarter. Under current and future climates, suitable habitats for the plant and the pest are concentrated in the Qinghai–Tibet Plateau, reaching their maximum under SSP245 and SSP126 in the 2090s, respectively. Current regions exclusively suitable for the plant without the pest are primarily found in eastern and central Tibet, reaching a maximum under SSP245 in the 2090s. Under climate change, the plant’s suitable habitats, free from the pest damage, are predicted to be concentrated in eastern Tibet and northwestern Yunnan. Our findings provide practical guidance for *F. unibracteata* cultivation, as well as the monitoring and prevention of *E. baileyi*.

## 1. Introduction

Medicinal plants are indispensable in both contemporary human medicine and traditional medicinal practices, with an estimated 75% of the global population depending on them for healthcare, particularly in developing countries [1]. These plants are crucial for public health and contribute significantly to socioeconomic advancement, especially in rural communities [2]. However, this critical resource is increasingly threatened by climate change, which disrupts habitat suitability, alters distribution patterns, and affects the phenology of medicinal plants [3,4]. Consequently, the survival of medicinal plants and the sustainability of their use face formidable challenges. Predictive studies have indicated that the suitable ranges for numerous medicinal plants will likely contract or shift significantly due to climate change [5,6,7]. While these plants may adapt by colonizing newly suitable habitats under future climatic conditions, the depletion of available medicinal resources may lead to considerable economic losses for local communities [8].

Higher temperatures accelerate pest development and population growth, while increased precipitation provides more abundant food resources, facilitating their proliferation and dispersion [9]. Therefore, climate warming may intensify pest pressures from insects, mites, and rodents, posing a significant threat to the sustainable utilization of medicinal plants, and hindering economic development in impoverished regions. Identifying suitable regions for medicinal plants and their pests is imperative to screen pest-free cultivation regions. Such strategies are essential to mitigate the adverse impacts of climate warming on medicinal plant production and safeguard the socioeconomic benefits of medicinal plant cultivation.

Fritillariae Cirrhosae Bulbus (FCB, known as Chuanbeimu in Chinese) is a highly valued Chinese medicinal material renowned for its therapeutic properties in heat clearance, knot dispersal, phlegm resolution, and cough suppression [10]. It is sourced from five species within the *Fritillaria* genus, with *F*. *unibracteata* Hsiao et K.C. Hsia (FU) being the principal contributor [11,12]. In recent years, the escalating demand for FCB, driven by economic incentives, has led to intensive harvesting of wild FU, resulting in a severe depletion of this medicinal resource [12]. Consequently, artificial cultivation has emerged as a pivotal approach to ensure the sustainable supply of FCB. However, FU is environmentally sensitive, requiring specific conditions such as high-altitude environments and shaded locations under shrubbery, and has a growth cycle exceeding three years. This complexity poses significant challenges to its cultivation and introduction. Therefore, identifying the critical environmental factors influencing FU distribution and its suitable habitats is significant for scientific cultivation.

The subterranean bulbs of FU are rich in protein and soluble sugars, making them an attractive food source for the plateau zokor, *Eospalax baileyi* Thomas (EB) [13]. Moreover, FU flourishes in loose soil, with its roots situated 5–15 cm beneath the surface, aligning with the feeding habits of the plateau zokor [14]. The overlap in habitat usage has detrimental effects, as EB not only consumes FU bulbs but also damages the plants by burrowing tunnels near the roots, resulting in plant mortality and substantial production losses [15]. In recent years, the damage caused by EB has escalated alongside the proliferation of FU cultivation [16]. Traditional control approaches, including the installation of subterranean iron wire mesh, application of chemical pesticides, and utilization of physical traps, are not only expensive but also exhibit restricted efficacy. Furthermore, overdependence on pesticides can lead to soil contamination, while implementing wire mesh or traps is resource-intensive and may not provide sufficient protection. Considering that FU requires a growth period exceeding three years before harvest, any inadequacy in executing timely preventative and control measures at any growth stage may lead to substantial yield losses or complete crop failure. Therefore, identifying and selecting suitable habitats for FU cultivation free from EB infestation are crucial to mitigate pest damage and ensure the production of this medicinal plant.

In recent years, species distribution models (SDMs) have emerged as potent tools for simulating and predicting species distribution, utilizing the occurrence records and available environmental data [17,18]. These models are widely applied in biological introduction and cultivation [19], species conservation [5], and pest management [20]. Among various SDMs, the maximum entropy model (MaxEnt) is notable for its high accuracy, reliability, computational efficiency, and operational flexibility [21,22]. Although the existing default settings in MaxEnt are derived from extensive empirical research, recent findings suggest that these defaults may result in model overfitting and suboptimal performance. Therefore, the existing predictions concerning the distribution of FU [23] and EB [24] might diverge from actuality. Moreover, the determination of suitable habitats for FU cultivation that are unaffected by EB infestation remains unclear.

In this study, MaxEnt with optimal settings was employed to predict the current and future distributions of FU and EB, and identify the dominant environmental factors influencing their distributions. Furthermore, ArcGIS was utilized to pinpoint the suitable regions for FU cultivation without EB damage. Our work provides critical insights for guiding the cultivation and production of FU, as well as the detection and control of EB.

## 2. Results

### 2.1. Screening of Distribution Records and Environmental Factors, and Accuracy of MaxEnt Prediction

After screening, 78 out of 124 distribution records and 15 out of 42 environmental factors were selected to predict FU distribution (Table 1). Consequently, 61 distribution records and 16 environmental factors were chosen to predict EB distribution (Table 1). The filtered distribution records of the two species were all concentrated in the Qinghai–Tibet Plateau of China. The results of Kuenm indicated that the optimal candidate model for FU distribution prediction was FC = LQ and RM = 1.9. Similarly, for EB distribution prediction, the optimal model was identified as FC = LPTH and RM = 4.0 (Figure S1).

The optimal MaxEnt models were utilized to simulate the current suitable habitats of FU and EB, respectively, based on their screened distribution records and environmental factors. The simulation results showed that the average training AUC and the TSS were 0.995 and 0.982 for FU prediction, and 0.997 and 0.952 for EB prediction, respectively, confirming the high reliability of the optimal models for subsequent predictions.

### 2.2. Critical Environmental Factors Affecting FU and EB Distribution

The critical environmental factors affecting the two species’ distributions were determined via the MaxEnt jackknife method. As illustrated in Table 1, the cumulative contributions and permutation importance of climate and elevation factors accounted for 96.7% and 99.9% of the FU distribution prediction, respectively, while those for EB were 95.9% and 98.3%. Our results indicated that climate and elevation were the determinants of the two species’ distributions. Among the selected climate and elevation factors, elevation (elev) and annual mean temperature (bio01) exerted a more pronounced influence on the distribution of FU, cumulatively contributing to 67.4%. Similarly, the elevation and precipitation of warmest quarter (bio18) exhibited a greater impact on the distribution of EB, with a cumulative contribution of 78.1%.

**Figure 1:**
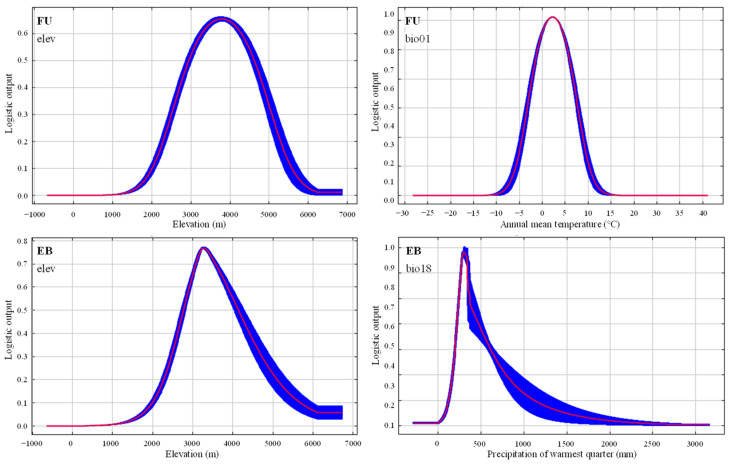
Response curves of environmental factors for FU and EB distribution prediction. The curves show the mean response of five replicates Maxent runs (red) and the mean ± one standard deviation (blue, two shades for categorical variables).

The relationships between the distributional probability and environmental factors were identified using single-factor response curves output by the MaxEnt model. The suitable ranges [habitat suitability index > MTSPS (0.1344)] of elev and bio01 for FU distribution prediction were 2117.62–5467.86 m and −5.15–9.68 °C, respectively. Similarly, the suitable ranges [habitat suitability index > MTSPS (0.2696)] of elev and bio18 for EB distribution prediction were 2442.08–5135.86 m and 191.72–660.10 mm, respectively (Figure 1).

### 2.3. Current Distribution of FU and EB

**Figure 2:**
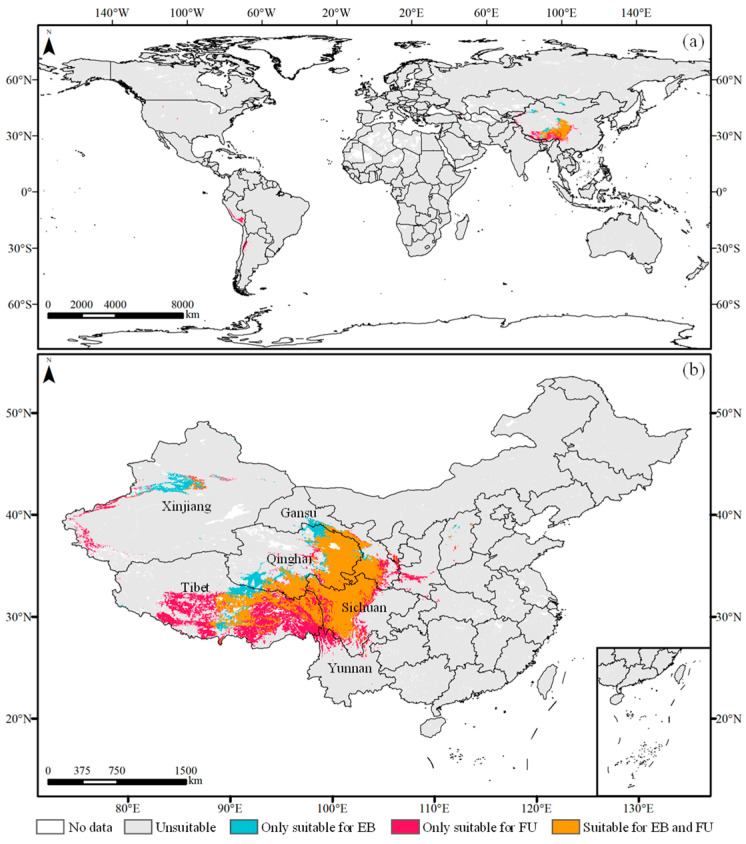
Current suitable habitats of FU and EB in the (**a**) world and in (**b**) China.

**Figure 3:**
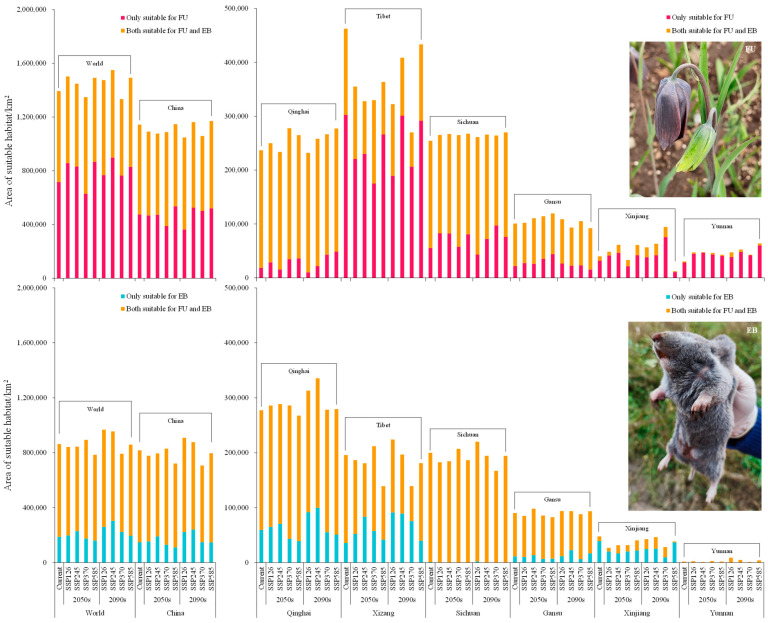
Area of suitable habitats for FU and EB distribution.

The optimal MaxEnt model was utilized to simulate the distribution of FU and EB based on the screened environmental factors and distribution records under current climatic conditions. As shown in Figure 2a and Figure 3, the suitable habitat areas for FU and EB in China covered 114.42 × 10^4^ km^2^ and 81.73 × 10^4^ km^2^, respectively, accounting for 82.14% and 94.59% of their global distributions. These habitats were mainly situated on the Qinghai–Tibet Plateau. Additionally, other countries adjacent to the Qinghai–Tibet Plateau, along with those in South America, including Peru, Chile, and Argentina, possessed smaller but suitable habitats for FU. The central region of Mongolia also encompassed limited but suitable habitats for EB.

The overlapping suitable habitats (both suitable for FU and EB, 67.65 × 10^4^ km^2^) were concentrated in the eastern Qinghai–Tibet Plateau (Figure 2b), primarily within Sichuan (19.91 × 10^4^ km^2^) and Qinghai (21.80 × 10^4^ km^2^) Provinces, followed by the Tibet (16.01 × 10^4^ km^2^) and Gansu (7.90 × 10^4^ km^2^) Provinces of China (Figure 3). The habitats exclusively suitable for FU in China, totaling 47.52 × 10^4^ km², were located in central and eastern Tibet (30.23 × 10^4^ km^2^) and central and western Sichuan (5.54 × 10^4^ km^2^), followed by Xinjiang (3.18 × 10^4^ km^2^), Yunnan (2.86 × 10^4^ km^2^), and Gansu (2.19 × 10^4^ km^2^) Provinces. Conversely, the habitats (18.76 × 10^4^ km^2^) exclusively suitable for EB were primarily situated near the western edge of the overlapping regions and central Xinjiang.

### 2.4. Future Distribution of FU and EB

**Figure 4:**
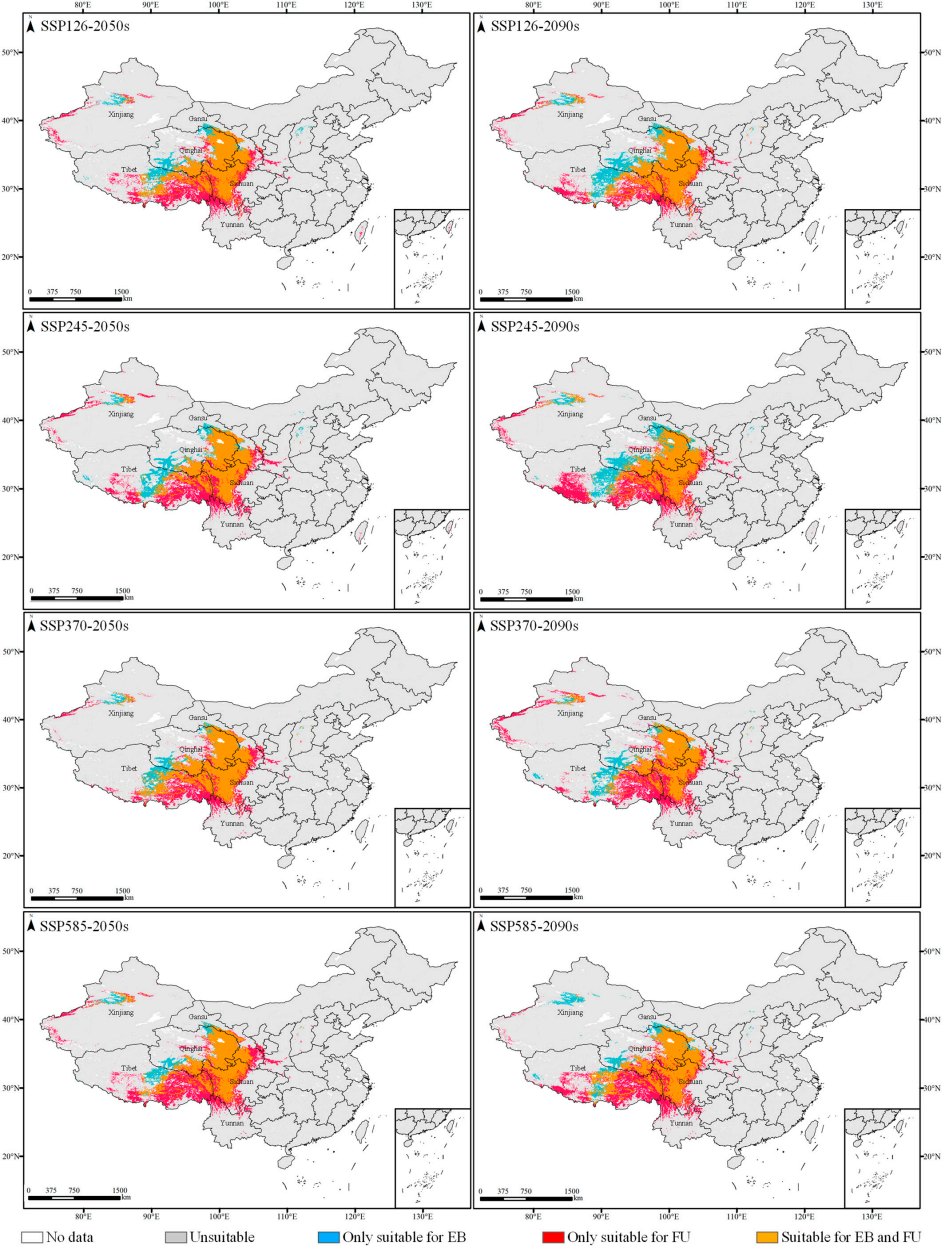
Suitable habitats for FU and EB distribution in the 2050s and 2090s under different climate scenarios.

Under future climate scenarios of the 2050s and 2090s, the global distribution of suitable habitats for FU and EB was anticipated to be consistent with the current habitats, predominantly situated in the southern and eastern Qinghai–Tibet Plateau (Figure 4 and Figure S2). However, the influence of climate warming on the distribution of the two species varied. In the 2050s and 2090s projection, the suitable area for FU distribution in the world was larger than the current ones, except for SSP370 in the 2050s and SSP370 in the 2090s, and reached its maximum under SSP245 in the 2090s (154.95 × 10^4^ km^2^) with a growth rate of 11.23% (Figure 3). For EB distribution, the suitable area reached the maximum under SSP126 in the 2090s (96.79 × 10^4^ km^2^), increasing by 12.02% compared to the current area. Conversely, the smallest suitable habitat area for EB was projected under SSP585 in the 2050s (79.22 × 10^4^ km^2^), decreasing by 9.10%. The overlapping suitable habitats for both species in the 2050s and 2090s were primarily situated in the eastern Qinghai–Tibet Plateau (Figure 4), mainly distributed in Sichuan and Qinghai Provinces, followed by Tibet and Gansu Provinces, China.

In China, the future habitats that were exclusively suitable for FU distribution without EB exceeded the current ones under SSP585 in the 2050s (53.58 × 10^4^ km^2^), SSP245 in the 2090s (52.53 × 10^4^ km^2^), SSP370 in the 2090s (50.10 × 10^4^ km^2^), and SSP585 in the 2090s (52.08 × 10^4^ km^2^), and reached the maximum under SSP585 in the 2050s, with an increase of 12.74% (Figure 3). Under SSP585 in the 2050s, the habitats only suitable for FU were concentrated in eastern and central Tibet (26.62 × 10^4^ km^2^); western, central, and southern Sichuan (8.10 × 10^4^ km^2^); eastern Gansu (4.40 × 10^4^ km^2^); and northwestern Yunnan (4.09 × 10^4^ km^2^). The habitats only suitable for EB achieved the maximum under SSP245 in the 2090s, increasing by 62.03% compared to the current ones, mainly distributed in Qinghai (9.98 × 10^4^ km^2^) and Tibet (8.93 × 10^4^ km^2^) Provinces.

### 2.5. Overlapping Habitats for EB Without FU Damage Under Climate Change

**Figure 5:**
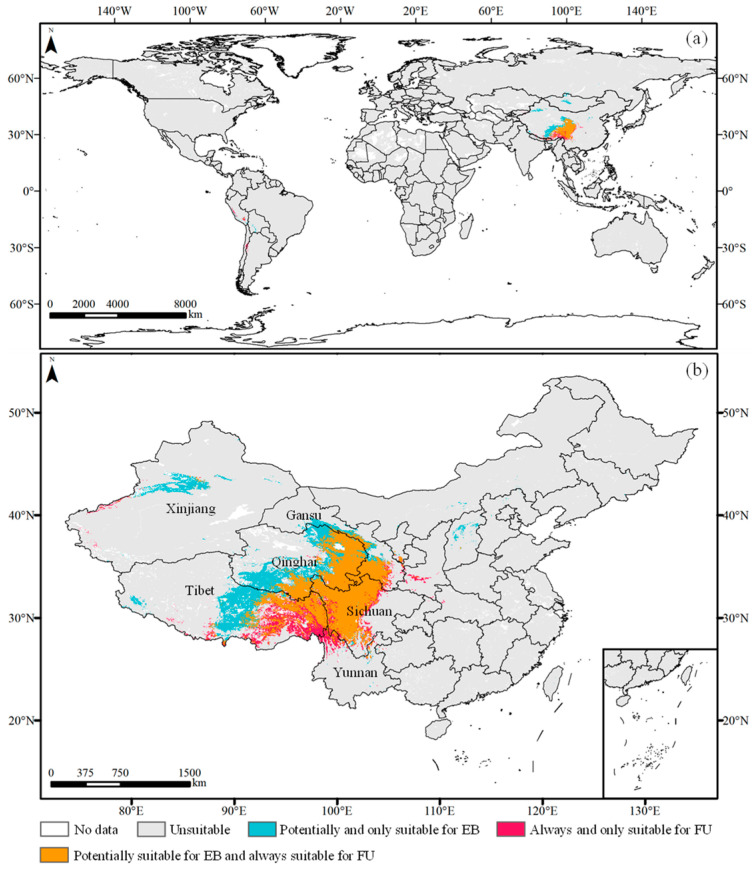
Overlapping habitats suitable for EB without potential FU in the (**a**) world and (**b**) in China across current and future climate scenarios.

Under current and future climate scenarios, the habitats (92.10 × 10^4^ km^2^) consistently suitable for FU distribution were concentrated in the northeastern, eastern, and southeastern Qinghai–Tibet Plateau of China, while the potentially suitable habitats (116.65 × 10^4^ km^2^) for EB were predominantly located in the northeastern, eastern, southeastern, and central Qinghai–Tibet Plateau. The habitats consistently suitable for FU, without potential EB damage, were concentrated in southeastern Tibet (10.52 × 10^4^ km^2^), central and western Sichuan (2.99 × 10^4^ km^2^), and northwestern Yunnan (2.23 × 10^4^ km^2^) (Figure 5).

## 3. Discussion

In this study, we utilized an optimized MaxEnt model to simulate and predict the potential distribution of FU and its pest EB, aiming to identify suitable habitats for FU that are free from EB damage under changing climatic conditions. Unlike previous studies that primarily focused on single-species distribution patterns [22] or species interactions without explicit spatial exclusion analysis [25], we integrated optimized MaxEnt with ArcGIS to identify non-overlapping suitable habitats for FU cultivation free from EB damage. This innovative dual-species exclusion approach directly addresses pest management needs, contrasting with conventional SDMs that emphasize habitat suitability alone. While Wang et al. [26] demonstrated the importance of local edaphic factors for specialist species, our elevation-driven framework was particularly effective for alpine species, where topographic factors supersede soil parameters in distribution determination. The methodology developed here, combining parameter-optimized niche modeling with geospatial conflict analysis, provides an adaptable framework for protecting high-value plants from specialized pests under climate change, particularly in topographically complex regions where traditional pest control methods are impractical.

### 3.1. Critical Environmental Factors Affecting Distribution

For various plant and animal species, soil is a fundamental but not the predominant factor affecting their growth and development [27]. Despite FU and EB preferring to inhabit soft soil, our results indicated that soil factors were not the primary determinant of their distribution. This observation has been widely documented across numerous plant and animal species [7,19,28]. For instance, Song et al. [20] found that while soil factors such as water content influenced the eclosion of the fruit fly, *Neoceratitis asiatica* (Becker), their contribution only accounted for 8.6%.

In this study, geographic factors, including elevation, slope, and aspect, were incorporated into the environmental factors to enhance the predictive accuracy of FU and EB distribution. We found that elevation was the most important environmental factor influencing the distribution of the two alpine species. Research has demonstrated that microtopography strongly affects mountain niches’ properties, with even small variations in microtopography significantly impacting soil temperature, freeze–thaw cycles, snow drifting, and wind patterns [29,30]. For this reason, geographic factors likely serve as proxies for elusive climatic factors. Oke and Thompson [30] demonstrated that excluding elevation as a predictor for alpine species led to a marginal decrease in model performance while simultaneously causing significant overestimates of species’ niche breadths.

Our results demonstrated that altitude was the most significant factor influencing the two species’ distribution, with suitable habitats ranging from 2442.08 m to 5135.86 m for EB and 2117.62 m to 5456.86 m for FU. Therefore, the altitudinal ranges exclusively suitable for FU were 2117.62 m–2442.08 m and 5135.86 m–5456.86 m. Cultivating FU within these specific altitudinal ranges could potentially mitigate EB damage. 

#### 3.1.1. Critical Environmental Factors Affecting EB Distribution

The exceptional oxygen-carrying capacity of EB can be attributed to elevated hemoglobin levels, a high intrinsic affinity for oxygen, and an abundant population of red blood cells [31]. These traits enable their respiratory and cardiovascular systems to efficiently cope with hypoxia and hypercapnia, enabling them to survive in high-altitude environments. Our results showed that EB was distributed at altitudes ranging from 2442.08 m to 5135.86 m, with habitat suitability vanishing outside this range. Previous studies have demonstrated that EB was exposed to increased oxygen concentrations at lower elevations, which impaired their physiological and metabolic processes, and ultimately threatened their survival [14,32]. Additionally, the scarcity of food resources further limits EB survival capacity at higher altitudes, as the diversity and abundance of plant species decrease with increasing elevation [33].

Our research indicated that EB survived in regions where precipitation ofwarmest quarter ranged from 191.72 mm to 660.10 mm, which aligned with Chu Bin’s findings. He has demonstrated that EB predominantly inhabited regions with an annual precipitation of 200 mm to 750 mm [34]. Given that the plateau zokor primarily feeds on plant roots, and that precipitation is essential for plant growth, adequate rainfall guarantees the zokor sufficient food resources to survive. However, excessive rainfall may compromise the structural integrity of EB’s burrows, thereby impeding its survival.

#### 3.1.2. Critical Environmental Factors Affecting FU Distribution

Plants inhabiting high altitudes exhibit remarkable adaptability to extreme conditions and can modify their morphological characteristics in response to environmental challenges [35]. Previous studies have demonstrated that FU’s plant height, bulb size, and leaf area decreased with increasing altitude [36,37], while its flower and seed weight were positively correlated with altitude [38]. These morphological alterations are considered adaptations to the alpine habitat rather than simple responses to adverse environmental conditions, allowing FU to thrive in high-altitude and low-temperature environments [39]. Our findings indicated that FU could survive at altitudes ranging from 2117.62 m to 5456.86 m, with an annual mean temperature of −5.15 °C to 9.68 °C. Altitudes and annual mean temperature outside these ranges were unsuitable for FU distribution due to the distinct climatic conditions. Elevated or depressed temperatures in these regions hinder FU growth, thereby threatening its viability. Li et al. [40] reported that FU’s growth slowed, its floral development was hampered, and bulb size progressively decreased at lower altitudes where temperature exceeded 30 °C. Conversely, the extremely cold temperature at higher elevations hinders the germination of FU seeds or bulbs.

### 3.2. Suitable Habitats for FU and EB Distribution

Our results indicated that these two alpine species were predominantly distributed across the Qinghai–Tibet Plateau of China under current climatic conditions, consistent with their observed distribution. The suitable habitats for EB were predominantly located in the northeastern, eastern, and central Qinghai–Tibet Plateau, which aligned with the findings of Wang et al. [33]. However, our study identified a larger area of suitable habitats for the plateau zokor than their results. Although the MaxEnt model was optimized in both studies, variations in the quantity and spatial distribution of species occurrence data led to differences in model optimization parameters and the criteria for delineating suitable habitats [41]. Consequently, these factors influenced the consistency of the prediction outcomes.

We found that the suitable habitats for FU distribution were concentrated in the northeastern, eastern, southern, and central Qinghai–Tibet Plateau, beyond the range identified by Zhao et al. [23]. This discrepancy may be attributed to differences in model parameters. Specifically, Zhao et al. utilized the MaxEnt model with default parameters, which might result in overfitted prediction results [42].

Under the current climatic conditions, habitats suitable for FU but unsuitable for EB were concentrated in eastern and central Tibet and northwestern Yunnan. Our findings, supported by other studies [41], suggested that these regions were not conducive to EB distribution. Therefore, we hypothesized that cultivating FU in these regions could prevent potential damage from EB. The findings of our field survey confirmed this theory as well. In 2023, we observed severe damage to FU caused by EB in Huzhu County (36°59′48″ N, 102°00′15″ E), Qinghai Province, and Mao County (28°46′10″ N, 99°13′56″ E), Sichuan Province. Conversely, no such damage was observed in Shangri-La County (27°32′00″ N, 99°50′06″ E), Yunnan Province, where conditions were exclusively suitable for FU. Under current and future climate scenarios (2050s and 2090s), our results indicated that the habitats exclusively and consistently suitable for FU distribution without potential EB damage were primarily located in southeastern Tibet and northwestern Yunnan. We suggest that cultivating FU in these regions is an effective way to mitigate EB damage.

Climate change is projected to result in elevated temperatures and altered precipitation patterns, which will affect species distribution [8]. However, altitude is identified as the primary factor shaping the distribution of alpine species [30], potentially explaining why future climate fluctuations do not induce substantial changes in the distribution of FU and EB. The impacts of climate change on species distribution are multifaceted, causing shifts in species ranges towards higher latitudes and elevations [43]. Alpine species, already adapted to cold and low-oxygen environments, will be compelled to migrate to higher altitudes as temperatures rise [44]. Our results indicated that suitable habitats for the two alpine species in China were projected to shrink in the future compared to their current ranges under most climate scenarios.

### 3.3. Limitations

Species distribution is affected not only by external environmental factors such as climate, soil, human activity, and geography, but also by their intrinsic adaptability and natural enemies [45,46]. Additionally, SDMs generally presume that species distribution records remain static for predicting distributions [20]. Consequently, the MaxEnt prediction results may deviate from the actual distribution. Future research should integrate these factors to enhance the accuracy of predictions for FU and EB distribution under changing climatic conditions.

## 4. Materials and Methods

### 4.1. Acquisition and Processing of Distribution Records and Environmental Data

**Figure 6:**
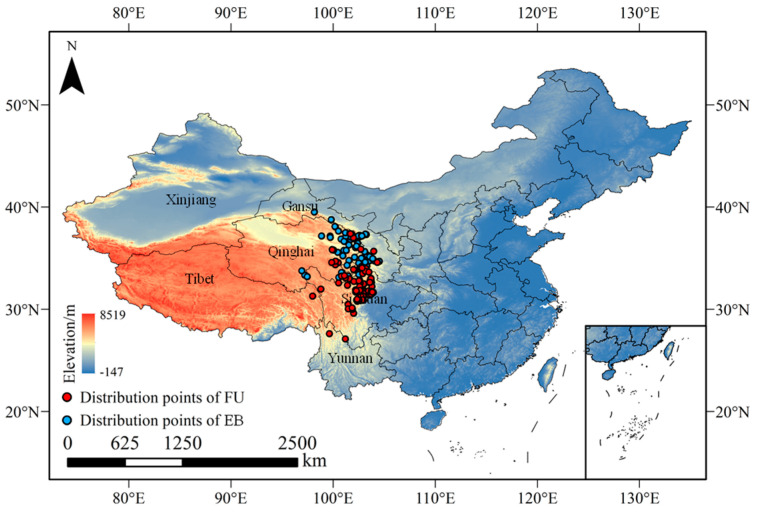
Distribution records of FU and EB in China.

The distribution records of FU were sourced from the Global Biodiversity Information Facility (GBIF) [47], the Flora of China (FOC, http://www.iplant.cn/foc/, accessed on 20 January 2024), China National Knowledge Infrastructure (CNKI, http://www.cnki.net/, accessed on 20 January 2024), and our investigation data collected from 2022 to 2023. The distribution records of EB were collected from CNKI and our investigative data from 2023. A total of 124 distribution records of FU and 90 distribution records of EB were obtained, all situated in China (Figure 6). Subsequently, the distribution records of the two species were examined separately utilizing the spatial analysis function of ArcGIS (v10.8, https://www.esri.com/zh-cn/arcgis/, accessed on 25 April 2023) to mitigate the impacts of spatial autocorrelation and enhance prediction accuracy. Only one point was retained when the distance between two records was less than 10 km.

Climatic data for the current situation (averages from 1970 to 2000), that in the 2050s (averages from 2041 to 2060), and that in the 2090s (averages from 2081 to 2100) were obtained from the Worldclim Database (v2.1, https://www.worldclim.org/, accessed on 16 January 2024). Future climatic data were determined based on the Beijing Climate Center Climate System Model (BCC-CSM) from the sixth phase of the Coupled Model Intercomparison Project (CMIP6), which included four climate scenarios based on the Shared Socioeconomic Pathways (SSP126, SSP245, SSP370 and SSP585) [48]. These scenarios represented a range of low to high greenhouse gas emissions, with each scenario encompassing 19 climatic factors (bio01–bio19). A digital elevation model (DEM) was acquired from the Geospatial Data Cloud (http://www.gscloud.cn/, accessed on 11 January 2024) to extract elevation, slope, and aspect factors. The topsoil data were downloaded from the World Soil Database (v1.2, https://www.fao.org/soils-portal/en/, accessed on 16 January 2024), which encompassed 17 soil factors. The human footprint data (hf_v2geo1) were sourced from the Global Human Disturbance dataset (http://sedac.ciesin.columbia.edu/wildareas/, accessed on 7 January 2024). The land cover (gm_lc_v3) and vegetation (gm_ve_v2) data were obtained from the Global Map data archives (https://globalmaps.github.io/, accessed on 7 January 2024). Above all, 42 environmental factors were collected and initially used to construct the MaxEnt model.

To mitigate overfitting of the MaxEnt model induced by multicollinearity among environmental factors, Pearson correlation among these environmental factors was analyzed using ENMTools (https://github.com/danlwarren/ENMTools/, accessed on 20 August 2023), and the contribution rates of these factors were calculated using the jackknife analysis of MaxEnt (v3.4.4, https://biodiversityinformatics.amnh.org/open_source/maxent/, accessed on 12 November 2023). Factors with zero contribution were removed, and only those with the highest contribution were retained when the absolute correlation coefficient between them exceeded 0.7.

### 4.2. Optimization, Construction, and Evaluation of MaxEnt

To mitigate overfitting of MaxEnt and enhance predictive accuracy, the Kuenm package (https://github.com/marlonecobos/kuenm/, accessed on 14 November 2023) in R (v3.6.3, https://www.r-project.org/, accessed on 11 December 2020) was employed to calibrate Feature classes (FCs) and regularization multipliers (RMs) to select the optimal combination for MaxEnt. The MaxEnt model has five distinct FCs, linear (L), quadratic (Q), hinge (H), product (P), and threshold (T), with 31 combinations. The combination of 40 RMs (ranging from 0.1 to 4.0 with an interval of 0.1) and 31 FC combinations was utilized to generate a total of 1,240 candidate models, and these models were evaluated using Kuenm based on the screened environmental factors. The optimal candidate model for MaxEnt was selected based on the following criteria: significant models with omission rates of ≤5% and the lowest delta-corrected Akaike information criterion (ΔAICc) values of ≤2%.

The remaining parameters of MaxEnt were designated as follows: ‘Create responsive curves’, ‘Do jackknife to measure factor importance’, ‘Out format: logistic’, ‘Random seed’, ‘Random test percentage: 25’, ‘Replicates: 5’, ‘Replicated run type: bootstrap’, ‘Write plot data’ and ‘Write background predictions’. The rest of the parameters were configured to default settings.

The optimal MaxEnt prediction’s performance was assessed by the area under the receiver operating characteristic curve (AUC) and the true skill statistic (TSS). The AUC and TSS values range from 0 to 1 and −1 to 1, respectively; values closer to 1 indicate superior model performance [49]. The MaxEnt prediction is considered highly reliable and excellent when the AUC exceeds 0.9 and the TSS surpasses 0.8 [19].

### 4.3. Classification and Area Calculation of Suitable Habitats

The prediction results output by the optimal MaxEnt were reclassified and visualized using ArcGIS. The average logistic threshold value of maximum training sensitivity plus specificity (MTSPS) output by MaxEnt was used to categorize these results into suitability [(habitat suitability index (HSI) > MTSPS] and unsuitability (HSI ≤ MTSPS).

The SDMtoolbox (v2.0, http://www.sdmtoolbox.org/, accessed on 20 April 2023) was employed to convert the prediction ASCII files into binary files using the MTSPS threshold, with unsuitability represented by 0 and suitability by 1. The ‘Plus’ function in Spatial Analyst Tools of ArcGIS was employed to identify overlapping habitats that were consistently suitable for FU, and all habitats that were potentially suitable for EB across current and future climate scenarios. Furthermore, it was applied to identify overlapping habitats exclusively suitable for FU without potential EB damage [7]. The proportion of each habitat was calculated based on its grid number, and the acreage of each habitat was calculated according to its grid proportion to China’s land area.

## 5. Conclusions

In this study, the MaxEnt model with optimal parameters was employed to simulate and predict the distribution of FU and EB, and their overlapping habitats under current and future climate scenarios were identified using ArcGIS. Our results indicated that elevation and annual mean temperature were the critical factors affecting FU distribution, whereas for EB, the determinants were elevation and precipitation of warmest quarter. Under current and future climate scenarios, suitable habitats for FU were predominantly located in the northeastern, eastern, southern, and southeastern Qinghai–Tibet Plateau, with the widest distribution observed under SSP245 in the 2090s. Concurrently, suitable habitats for EB were concentrated in the northeastern, eastern, and central Qinghai–Tibet Plateau, reaching their maximum under SSP126 in the 2090s. The suitable habitats for FU free from EB reached their maximum distribution under SSP585 in the 2090s. Habitats consistently suitable for FU without potential EB interference were concentrated in southeastern Tibet and northwestern Yunnan. Our findings contribute to the cultivation of FU, and the monitoring and prevention of EB, thereby ensuring the cultivation benefits.

## Figures and Tables

**Table 1:** Environmental factors selected to construct the MaxEnt model for predicting the distributions of EB and FU.

Environmental Factors	Description	Percent Contribution (%)	
		FU	EB
aspect	Aspect	0.2	<0.1
bio01	Annual mean temperature	17.7	1.4
bio04	Temperature seasonality (standard deviation × 100)	7.7	4.6
bio14	Precipitation of driest month	3.3	4.4
bio15	Precipitation seasonality (coefficient of variation)	0.7	–
bio18	Precipitation of warmest quarter	5.9	10.9
bio19	Precipitation of coldest quarter	4.5	7.4
elev	Elevation	49.7	67.2
gm_lc_v3	Land cover	0.3	0.1
gm_ve_v2	Vegetation (percent tree cover)	2.5	–
hf_v2geo1	Human footprint index	0.2	3.7
slope	Slope	7.2	<0.1
t_caco3	Topsoil calcium carbonate	–	<0.1
t_caso4	Topsoil gypsum	–	0.3
t_cec_soil	Topsoil CEC (soil)	–	<0.1
t_ece	Topsoil salinity (Elco)	–	<0.1
t_esp	Topsoil sodicity (ESP)	<0.1	–
t_oc	Topsoil organic carbon	–	<0.1
t_teb	Topsoil TEB	0.2	–
t_texture	Topsoil texture	<0.1	<0.1

## Data Availability

Data will be made available on request.

## Supplementary Materials

The following supporting information can be downloaded at https://www.mdpi.com/article/10.3390/plants14050674/s1. Figure S1: Omission rates at 5% and AICc values for all candidate models for (a) FU and (b) EB distribution prediction; Figure S2: Future suitable habitats of FU and EB in the world.
