# Supplementary material for: Identifying Suitable Regions for Fritillaria unibracteata Cultivation Without Damage from the Pest Eospalax baileyi
Source: Plants (Basel). 2025 Feb 22;14(5):674. doi: 10.3390/plants14050674 (PMC11901731; doi:10.3390/plants14050674)
Supplement: Supplementary file 1 [file plants-14-00674-s001.zip › plants-3441844-supplementary.pdf]

Supplementary Materials

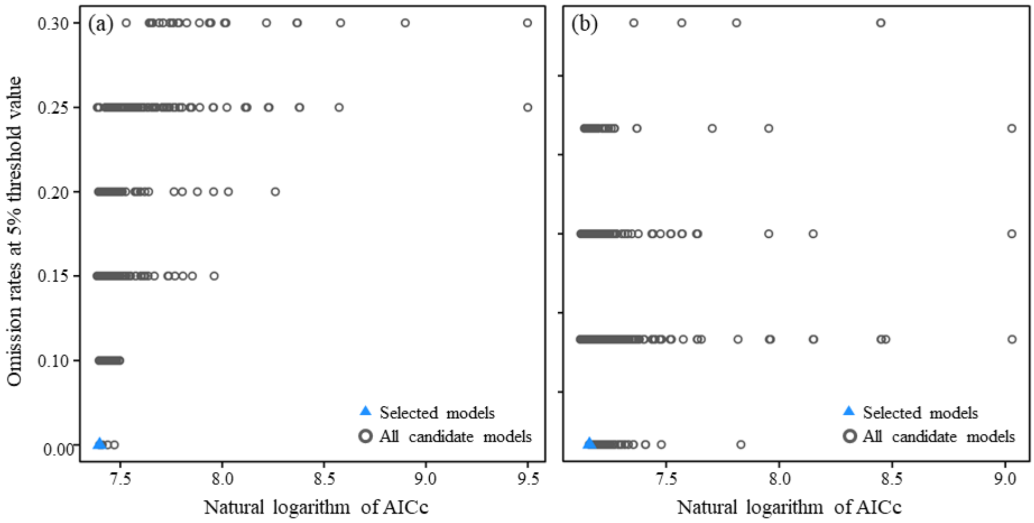

Figure S1. Omission rates at 5% and AICc values for all candidate models for (a) FU and (b) EB distribution prediction.

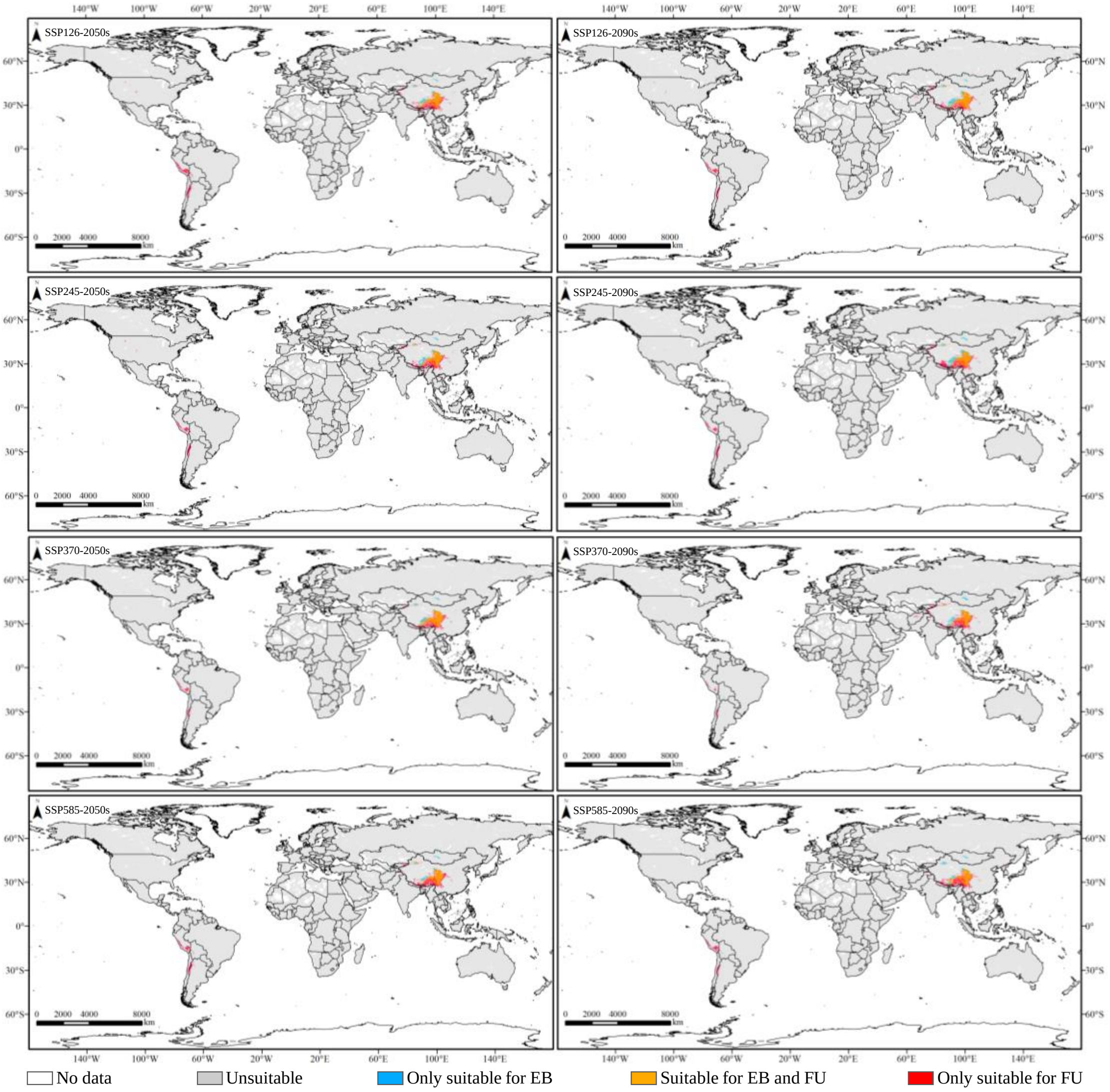

Figure S2. Suitable habitats for FU and EB distribution in the world under future 2050s and 2090s climate scenarios.
